# Toward a universal theory of consciousness

**DOI:** 10.1093/nc/niae022

**Published:** 2024-05-31

**Authors:** Ryota Kanai, Ippei Fujisawa

**Affiliations:** President Office, Araya, Inc., Sanpo Sakuma Building, 1-11 Kanda Sakuma-cho, Chiyoda-ku, Tokyo 101-0025, Japan; President Office, Araya, Inc., Sanpo Sakuma Building, 1-11 Kanda Sakuma-cho, Chiyoda-ku, Tokyo 101-0025, Japan

**Keywords:** theories of consciousness, universality, global workspace, integrated information theory, higher-oder theory

## Abstract

While falsifiability has been broadly discussed as a desirable property of a theory of consciousness, in this paper, we introduce the meta-theoretic concept of “Universality” as an additional desirable property for a theory of consciousness. The concept of universality, often assumed in physics, posits that the fundamental laws of nature are consistent and apply equally everywhere in the universe and remain constant over time. This assumption is crucial in science, acting as a guiding principle for developing and testing theories. When applied to theories of consciousness, universality can be defined as the ability of a theory to determine whether any fully described dynamical system is conscious or non-conscious. Importantly, for a theory to be universal, the determinant of consciousness needs to be defined as an intrinsic property of a system as opposed to replying on the interpretation of the external observer. The importance of universality originates from the consideration that given that consciousness is a natural phenomenon, it could in principle manifest in any physical system that satisfies a certain set of conditions whether it is biological or non-biological. To date, apart from a few exceptions, most existing theories do not possess this property. Instead, they tend to make predictions as to the neural correlates of consciousness based on the interpretations of brain functions, which makes those theories only applicable to brain-centric systems. While current functionalist theories of consciousness tend to be heavily reliant on our interpretations of brain functions, we argue that functionalist theories could be converted to a universal theory by specifying mathematical formulations of the constituent concepts. While neurobiological and functionalist theories retain their utility in practice, we will eventually need a universal theory to fully explain why certain types of systems possess consciousness.

## Introduction

Consciousness, one of the most enigmatic and debated phenomena in science, has been a subject of numerous theories and propositions (Seth and Bayne 2022). Each theory attempts to unravel what makes an entity conscious. Recently, the field has witnessed heated debates about testing these theories (Lenharo 2023). The Cogitate project (Melloni et al. 2021, 2023), for instance, embarked on an ambitious collaborative project to directly compare the global workspace theory (GWT) (Baars 1993, Dehaene et al. 1998, Dehaene and Changeux 2011, Mashour et al. 2020, Baars et al. 2021, VanRullen and Kanai 2021) and integrated information theory (IIT) (Oizumi et al. 2014; Tononi et al. 2016, Albantakis et al. 2023) using empirical tests in adversarial collaboration. While the project’s aim was commendable, its results presented a myriad of challenges (Lau 2023). A central issue was whether these theories, particularly IIT, were ripe for falsification—a hallmark of scientific inquiry as championed by thinkers like Popper (Popper 2005). Falsifiability has been a significant point of contention in consciousness research (Kleiner and Hoel 2021), with IIT often at the center of such debates (Doerig et al. 2019). In this context, a more nuanced approach to evaluating scientific progress such as Lakatos’ concept of scientific research programs (Lakatos 1969) has been also discussed (see Negro 2020).

The criteria for the scientific status of theories of consciousness should not be confined solely to falsifiability. We contend that a theory of consciousness should possess other meta-theoretic attributes. Notably, theories such as IIT stand out for their predictive prowess beyond merely brain-centric systems—a trait conspicuously absent in many other consciousness theories. To encapsulate this essential quality, we introduce the notion of “Universality” as an additional criterion to assess theories of consciousness. This paper discusses how this criterion relates to prevailing theories of consciousness. While we argue that many current theories fall short of this benchmark, we also envision pathways for them to embrace universality.

## Universality

In the landscape of scientific theories, certain criteria stand out as essential benchmarks for the validity and applicability of theories (see Doerig et al. 2021). One such criterion, which we propose as pivotal for theories of consciousness, is that of “Universality.” (Doerig et al. (2021) outline four hard criteria for consciousness theories, with their fourth criterion referring to the ability of a theory to cope with the multiple realization of functions across diverse entities. This closely parallels the concept of universality we explore in our work. However, our universality criterion is somewhat more stringent. For example, while Doerig et al. (2021) consider theories like GWT, Predictive Processing Theory, and Adaptive Resonance Theory to meet their fourth criterion, we argue that these theories, in their current forms, do not fully satisfy our universality criterion, which demands broader applicability across all dynamical systems and a clear definition of consciousness constituents.) The concept of universality, often assumed in physics, posits that the fundamental laws of nature are consistent and apply equally everywhere in the universe, and they remain the same over time. This is a crucial concept in science, acting as a guiding principle for developing and testing physical theories. The belief in universality means that the knowledge and insights we gain from experiments on Earth are applicable to the entire universe. This property is highly desirable in any theory of physics because it provides a level of reliability and predictability, allowing scientists to make confident predictions beyond what we can directly observe. The pursuit of universal laws is essentially a pursuit of the underlying, unchanging rules that govern all of nature.

Similarly, the attribute of universality is highly desirable in developing a theory of consciousness. A universal theory of consciousness would propose fundamental principles and laws that account for conscious experiences in any entity, irrespective of its physical composition or origin. This universality is desirable as it would allow for a comprehensive understanding of consciousness, since it would enable researchers to generalize findings and make predictions about consciousness beyond what we can easily observe, namely reports of consciousness in humans and close species. Such a theory would not only illuminate the nature of human consciousness but also provide insights into the potential existence and characteristics of consciousness in other forms of life and artificial entities, bridging the gap between the physical and the experiential realms.

We define universality as the ability of a theory to determine whether a given dynamical system is conscious, irrespective of its origin or composition (e.g. whether it is biological brain, hurricane or computers). This means that the theory must be applicable to any physical system as long as its dynamics is fully described with, say, a transition probability matrix or a Langevin equation. Since physical systems can be described at multiple scales, dynamics needs to be described at a granularity level at least as detailed as the level concerned by the theory under consideration. This assumption is made to ensure that the relevant features are detectable if present in the target physical system.

What aspects of consciousness a theory should predict is also important when we consider universality. The primary target is the presence of consciousness within a system. A theory should be able to tell which part of the system corresponds to a conscious system as opposed the environment. In the case of the biological brain, a theory must be able to predict which set of neurons realizes conscious experience, and which part does not.

Additionally, the next target of prediction is the qualitative aspect of consciousness. Once the theory manages to successfully identify the parts of the system that realize consciousness, it is desired that the theory makes predictions about the quality of conscious experience. For example, this could be about whether the conscious experience of the system is more like vision or audition (von Melchner et al. 2000, Kanai and Tsuchiya 2012). Once this is achieved, one could address the famous question of what it is like to be a bat (Nagel 1980) by showing the experience of sensing the spatial environment with echolocation is more like seeing or hearing (Tsuchiya 2017).

The impetus behind the universality stems from the premise that consciousness also obeys certain laws of nature. As such, it should not be bound by the specifics of human biology or Earth’s evolutionary history. If certain physical conditions are met, consciousness could, in theory, emerge in non-biological systems, artificial intelligences, or even extraterrestrial entities with entirely different evolutionary trajectories. A truly comprehensive theory of consciousness should, therefore, be able to address these diverse manifestations. If the ultimate aim of a theory of consciousness is to tackle the Hard Problem, then the theory must extend beyond the confines of the biological brain. A theory that merely predicts which regions of the primate brain correlate with consciousness is inherently limited. It would fall short in elucidating the “why,” “how,” and under which conditions consciousness arises from a physical entity. This underscores our conviction that the universality criterion is indispensable for the evolution of a comprehensive theory of consciousness.

Theories in physics possess the characteristic of universality. The laws of physics, once discovered and validated in one part of the universe, are expected to hold true across varied locations and times. This consistency and ubiquity underscore the very essence of laws of natural sciences. In the annals of physics, there was once a belief that the laws governing Earth differed from those that ruled celestial bodies. Newton’s groundbreaking realization was that both terrestrial and celestial realms obeyed the same universal laws of physics. This revelation of universality illustrates that the strength of theories lies in their ability to be conceived and tested within our immediate realm yet remain applicable to phenomena beyond our direct reach or measurement.

Such potential for universality is not only a testament to the elegance of physics but also a cornerstone of its success as a scientific discipline. Similarly, for theories of consciousness to be truly comprehensive and effective, they too should embody this principle of universality, ensuring their applicability across a diverse range of entities and scenarios.

Drawing a parallel to consciousness research, a fundamental theory of consciousness should exhibit a similar universality. Just as physical laws do not change from one galaxy to another, a theory crafted and tested for consciousness as we know it—primarily within the human brain—should be equally applicable to other species and even non-biological systems. Consciousness, being a natural phenomenon, arises when specific physical conditions are met. Therefore, any comprehensive theory of consciousness should transcend the limitation of our immediate understanding, offering predictions about the existence of conscious experiences across diverse physical systems.

## Limitations of current theories of consciousness

While many theories in consciousness research offer valuable insights into specific aspects or manifestations of consciousness, they often do so within a limited scope, primarily focusing on human or mammalian consciousness. The universality criterion challenges these theories to broaden their horizons, to consider consciousness in its myriad potential forms, and to offer predictions that can be tested across a wide range of systems.

Now let us consider whether the universality criterion is met by some of the current theories. In this section, we will discuss prominent theories, IIT, GWT, and the higher-order theory (HOT), to elucidate these constraints.

### Integrated information theory

IIT is one of the few theories that satisfy the universality criterion. It is in principle applicable to any (discrete) dynamical system. It delineates which part of the system corresponds to a conscious entity separated from other entities or the environment. Regarding the second desiderata, IIT could in principle be used to identify the structure that corresponds to vision or hearing. While it remains challenging to actually compute constructs of IIT in practice for real complex systems such as the brain, the structure of IIT meets the universality criterion. It is worth noting that IIT is not the only theory that satisfies this criterion (e.g. information closure theory could also identify the conscious entity (Chang et al. 2020)). Also, as we discuss later, other theories could potentially be re-formulated as a universal theory.

### Global workspace theory

GWT posits that consciousness arises from the widespread sharing of information across various brain networks. When specific information becomes globally available, it enters our conscious awareness. GWT is primarily rooted in the understanding of the human brain. Its principles, while robust within this context, may not easily extend to non-biological or radically different biological systems (Carruthers 2018a, 2018b, Birch 2022). For example, in the current form of the theory, it is difficult whether an artificial intelligence system possesses a global workspace (Butlin et al. 2023).

For GWT to satisfy the Universality criterion, it needs clear mathematical definitions for each theoretical components such as global workspace, broadcasting, and ignition. What exactly constitutes a “global workspace” in mathematical or system terms? Without this clarity, GWT is not applicable to diverse systems. To satisfy the Universality criterion, we need to be able to say whether a particular system possesses a global workspace and what constitutes broadcasting and so on.

While we believe it should in principle be possible to convert GWT into a universally applicable theory, it is crucial to distinguish between different versions of GWT. The original formulation of GWT, as proposed by Baars (1993), primarily emphasizes the computational aspects of the global workspace. This approach centers on the algorithmic processes associated with consciousness, and thus universality could be built upon such computational algorithms. For this version of GWT, we would need to focus on how to define the algorithmic processes within a physical system. On other hand, the global neuronal workspace developed by Dehaene et al. (1998) and Mashour et al. (2020) shifts the focus to the neurobiological implementations of the global workspace. Here, the potential barrier to universality lies in providing a universally applicable definition for currently neurobiologically defined concepts. Furthermore, the recent conscious turing machine proposed by Blum and Blum (2022) offers another variation. It presents a computational architecture akin to GWT but does not rely on specific implementation.

Thus, when considering the conversion of GWT into a universal theory, it is important to take into account these distinct versions and their respective implications. Each presents unique challenges in defining the key concepts as a universal feature applicable to both biological and artificial systems.

### Higher-order theory

The HOT offers to elucidate what differentiates a conscious mental state from an unconscious one, particularly in cases where there is a dissociation between performance and subjective report, such as in blindsight phenomena. At its core, HOT proposes that there are distinct stages for first-order processes, which drives performance and higher-order processes for conscious report. This framework captures the essence of “awareness” as a form of access to primary information.

While the overarching premise of HOT is consistent, the theory itself has manifested in various forms (Lau and Rosenthal 2011). HOT suggests that consciousness arises when a cognitive system possesses a higher-order representation of its own mental states. From the perspective of the universality criterion, it remains vague what it means to have a higher-order representation or meta-representation unless we have a rigorous way to define meta-representations so that we can determine whether a given dynamical system contains a meta-representation or not (Butlin et al. 2023).

The difficulty of detecting higher-order representations in a non-brain system becomes apparent when we consider whether deep neural networks possess higher-order representations. A naïve interpretation might suggest that any transformation qualifies as higher-order (meta-)representation. For instance, if a meta-representation *y* of a first-order representation *x* is merely a transformation, *y *= *f*(*x*), then every subsequent layer in a neural network becomes a meta-representation of its predecessor. Such a broad definition dilutes the significance of meta-representation, making it far from the coveted “holy grail” of consciousness and inadvertently endorsing a form of panpsychism. Another perspective is uncertainty estimation. The correlation between confidence and performance in a primary task is frequently used as evidence of awareness in cognitive neuroscience (Kanai et al. 2010, Sandberg et al. 2010, Fleming et al. 2012, Fleming and Lau 2014). This implies that estimating uncertainty could be a form of meta-representation. In deep learning contexts, this might be achieved through rather simple mechanisms like softmax operation over the output layer. This too seems rather simple to capture the essence of consciousness, rendering most neural networks used in computer vision conscious.

While the mathematical definition of meta-representation remains elusive, a comprehensive exploration of this topic is beyond the scope of this paper. Here, we raised this as an example to illustrate the difficulty of finding a good definition that applies to a broader range of systems beyond human and animal behavioral paradigms. While both GWT and HOT offer valuable insights into the nature of consciousness, their current formulations seem tethered to human-centric perspectives. To truly embrace the universality criterion, these theories, among others, need to incorporate rigorous mathematical definitions applicable to a spectrum of systems.

## Functionalist theories can be made universal

Given our discussion so far, where IIT was presented as an exemplar of a theory embodying Universality in contrast to functionalist theories like GWT and HOT, one might be tempted to view this distinction as rooted in metaphysical differences. However, this is not our intention. We posit that functionalist theories have the potential to be made universal, if more rigorous definitions are provided for their constituent concepts.

At its heart, functionalism is predicated on the idea that mental states are constituted solely by their functional role, meaning their causal relations with other mental states, sensory inputs, and behavioral outputs. This perspective, in principle, is not restricted to human cognition or biological systems. It can be applied to any system that exhibits the requisite causal relations, be it a machine, an alien life form, or a complex network.

For example, the building blocks idea of consciousness proposes that consciousness arises when a set of key functions are instantiated in a system (Tait et al. 2023). This is a generalizable theory in the sense that any entity that satisfies those conditions is considered conscious. Thus, a functionalist theory does not have to be constrained to a neurobiological study and can potentially be extended beyond the brain.

## Intrinsic property

A pivotal challenge to render functionalist theories universal is to define those concepts as an intrinsic property of the system (not to be confused with the intrinsic perspective in IIT literature). Here, the term “intrinsic” denotes that the property is inherent to the system, independent of external interpretation but is instead defined as a property possessed by the system. For instance, the presence of recurrence through feedback can be considered an intrinsic property of the system. Reflecting on how various concepts integral to major functionalist theories can be defined intrinsically would be a constructive step forward.

Concepts such as “global workspace” or “higher-order representation” are understood by human neuroscientists. However, deciding whether they are present in an arbitrary physical system requires more precise mathematical definitions to allow their identification. It is important to note that this does not imply that global workspace or higher-order representations are inherently non-intrinsic properties of a system. Rather, the challenge lies in operationalizing these concepts in a manner independent of human-centric interpretations, thereby those concepts to be identified and measured in a broader range of systems. This perspective aligns with our argument in the previous section, where we emphasize the potential of functionalist theories to be extended to more universally applicable frameworks. Operationalizing global workspace and higher-order representations is a crucial step for refining those theories.

There have been several proposals to implement the functional building blocks of consciousness using deep neural networks (VanRullen and Kanai 2021, Juliani et al. 2022a, 2022b, Butlin et al. 2023). A functional theory of consciousness could be used as a way to determine whether a given system is conscious based on the functional properties of the system. However, for such a theory to attain universality, it is crucial that these functions are described in a precise way that does not require a judgment call, ensuring that assessments of consciousness are not contingent on the interpretations of an external observer.

## Extrapolation and panpsychism

Once a universal theory of consciousness is formulated, we can first test its validity in systems where the presence of consciousness is largely undisputed, such as humans and certain animals. After accumulating substantial empirical support for a specific theory, it becomes plausible to extrapolate the theory to non-biological or non-brain-based systems, making informed inferences about the presence of consciousness in artificial intelligences or extraterrestrial beings. While the consciousness of such entities remains unobservable and speculative, drawing inferences from well-supported theories is a foundational practice in science.

For instance, we infer the existence and characteristics of exoplanets, the composition of the Earth’s core, the properties of dark matter, and the behaviors of subatomic particles, all of which are not directly observable but are inferred through the lens of well-established theories and indirect observations. Although consciousness poses unique challenges due to its subjective nature and our inability to directly observe it in entities other than ourselves, the process of making informed inferences about unobservable systems is a common thread running through various scientific disciplines. The development and refinement of a universal theory of consciousness would thus enable us to extend our understanding of consciousness beyond the confines of our immediate experience, potentially unveiling the mysteries of consciousness in entities vastly different from ourselves.

However, it should be noted that a universal theory of consciousness does not imply panpsychism. Although a universal theory renders every physical system a potential target, it does not necessarily imply that every physical system is deemed conscious. Whether a given system should be regarded as conscious ultimately depends on the specifics of the theory applied. Furthermore, some theories might not simply categorize consciousness in a binary fashion but might quantify the degree of consciousness, perhaps on a continuum like real numbers.

On a related note, while IIT, which we deemed universal, is often associated with panpsychism (Tononi and Koch 2015), recent interpretations suggest viewing IIT as an emergentist theory (Cea 2021, Negro 2022). While IIT could attribute consciousness to non-biological systems, its attribution is graded, and many systems of different scales are indeed considered non-conscious according to IIT. Thus, universality does not necessarily imply panpsychism.

## The Specificity Problem in applying consciousness theories to non-humans cases

The challenge of applying human-centric theories of consciousness to non-human entities has been a recurring theme in philosophical discourse (e.g. Block 2002, Shevlin 2021, Birch 2022). Central to this discussion is the Specificity Problem, as articulated by Shevlin (2021), which is highly relevant for our current discussion on universality in consciousness theories.

The Specificity Problem refers to the difficulty in precisely defining cognitive mechanisms that constitute consciousness, in a way that is not only applicable to humans but also extends to non-human and non-biological systems (Shevlin 2021). In our view, this problem stems from the inherent ambiguity in many current theories of consciousness when extrapolated to such entities. Shevlin (2021) discussed the potential utility and limitations of the so-called theory-heavy and theory-light approaches (see Birch 2022).

Our approach would be categorized as a theory-heavy approach, which aims to discern non-human and machine consciousness by evaluating the presence of crucial mechanisms that realize consciousness according to our best theory of human consciousness. Although Shevlin indicates that such an approach might encounter the Specificity Problem, we propose that the notion of universality we proposed here may offer a potential remedy.

The Specificity Problem is framed in terms of cognitive theories that adopt high-level cognitive functions such as working memory, attention, and metacognition as the benchmarks for consciousness. These functions can be realized in multiple ways across biological and artificial systems and may exhibit varied performance levels. This variability introduces a degree of uncertainty in applying theories based on these cognitive functions to non-human cases.

The concept of universality that we advocate offers a potential solution to the Specificity Problem by emphasizing the need to clearly define causal mechanisms that are identifiable across various dynamic systems. This approach shifts our focus from abstract, high-level constructs to more concrete, underlying processes that can be operationally identified as indicators of consciousness. By doing so, universality broadens the applicability of consciousness theories beyond human-centric cases. Furthermore, by reinterpreting cognitive constructs as intrinsic properties within any physical system, we can transform existing cognitive theories, which are currently limited by the Specificity Problem, into their universal equivalents. This redefinition enables these theories to be applicable in a wider range of contexts, including non-human cases, through a theory-heavy approach.

## Is universality required for all theories of consciousness?

In this paper, we propose universality as an essential property for theories of consciousness. It is important to clarify, however, that we do not assert universality as a mandatory attribute for all scientific theories. Many theories, especially those in fields like psychology or sociology, are valuable and effective without being universal. This also holds true for certain types of theories of consciousness. For instance, theories that are not universal can be useful in applied contexts, such as predicting whether patients under anesthesia experience conscious pain. Universality becomes essential when the ambition of a theory extends to elucidating the fundamental link between consciousness and physical systems—which lies at the heart of the Hard Problem of consciousness. Thus, the necessity of universality in theories of consciousness depends on their goals. Our stance is that while not all theories require universality, universality gains importance when addressing the Hard Problem of consciousness and the even more challenging Harder Problem of attributing consciousness to non-biological systems (Block 2002).

## Universality and materiality in consciousness theories

So far, we argued that the advantage of universality is that it enables us to make theoretically informed inferences about the presence of conscious experience inside distant species and non-biological systems. In the current paper, we argued that functional roles underlying conscious mental states could be applicable to non-biological systems. This statement assumes independence of functions from their underlying medium. Admittedly, in our argument, we had the assumption that the functions here are computational processes, and thus it should be medium independent. However, this perspective contrasts with the views of several theorists who emphasize the crucial role of biological materiality in consciousness (e.g. Searle 1980, 1984, Block 2002, Revonsuo 2006, Aru et al. 2023), where the biological composition of the brain, particularly its neuronal and cellular structures, is integral to the realization of consciousness.

While our proposal of universality partially is built upon the assumption in line with computational functionalism or mechanistic functionalism (Piccinini 2020), the notion of universality is also relevant to theories that attribute a central role to the material properties of life for consciousness. For example, if a theory claims that consciousness is exclusively a feature of systems composed of biological neurons, universality remains applicable provided the theory offers clear criteria for identifying what constitutes “neurons” in physical systems. Such criteria would enable the application of the theory to assess whether various physical systems meet the defined conditions to be neurons. This approach underscores that universality, as a property of consciousness theories, can embrace both medium-independent and materiality-focused perspectives.

## Conclusions

In conclusion, we proposed the notion of universality as an additional criterion to characterize the nature of a theory of consciousness. The universality criterion serves as both a challenge and a guidepost for consciousness research, pushing the boundaries of current theories and pointing the way toward a more comprehensive understanding of consciousness in all its forms. The potential of formulating functionalist theories to be universal is an exciting future direction. By refining and rigorously defining their core concepts, these theories can begin to address consciousness in diverse systems beyond the brain-centric perspective.

The aim of this paper is not to assert the superiority of IIT over other theories but to underscore its unique fulfillment of the universality criterion, a rarity among existing theories of consciousness. While various theories of consciousness have been proposed to elucidate diverse facets of consciousness (Seth and Bayne 2022), they can yield valuable insights even without adhering to the universal criterion. Nonetheless, by delineating differences through the lens of universality, we aspire to clarify why IIT often diverges from and may seem counterintuitive compared to other prevailing theories.

Should other theories undergo rigorous mathematization of their pivotal concepts, capturing them as intrinsic properties of a system, we anticipate that they might appear more similar to IIT, facilitating more direct comparisons. While neurobiological and extrinsic functionalist theories retain their utility, the pursuit of a comprehensive understanding of consciousness necessitates the development of a universal theory, and we will eventually need a universal theory to fully explain why certain types of systems possess consciousness while others do not.
